# Costs and scale-up costs of community-based Oral HIV Self-Testing for female sex workers and men who have sex with men in Jakarta and Bali, Indonesia

**DOI:** 10.1186/s12913-024-10577-0

**Published:** 2024-01-22

**Authors:** Wayan Citra Wulan Sucipta Putri, Luh Putu Sinthya Ulandari, Ivy Cerelia Valerie, Bagus Rahmat Prabowo, Donny Hardiawan, Estro Dariatno Sihaloho, Riki Relaksana, Brigitta Dhyah Kunthi Wardhani, Ngakan Putu Anom Harjana, Nur Wulan Nugrahani, Adiatma Yudistira Manogar Siregar, Pande Putu Januraga

**Affiliations:** 1 Department of Public Health and Preventive Medicine, Faculty of Medicine, Udayana University, Jl. P. B. Sudirman, Denpasar, Bali 80232 Indonesia; 2 Center for Public Health Innovation (CPHI), Udayana University, Denpasar, Bali Indonesia; 3 UNAIDS Indonesia, Jakarta, Indonesia; 4 Center for Economics and Development Studies (CEDS), Department of Economics, Faculty of Economics and Business, Universitas Padjadjaran, Bandung, West Java Indonesia

**Keywords:** Costs, Cost analysis, Scale-up, HIV self-testing, Oral fluid test, Key populations, Knowledge of HIV status, Diagnosis, Screening, Indonesia

## Abstract

**Background:**

The proportion of individuals who know their HIV status in Indonesia (66% in 2021) still remains far below the first 95% of UNAIDS 2030 target and were much lower in certain Key Populations (KPs) particularly Female Sex Workers (FSW) and Male having Sex with Male (MSM). Indonesia has implemented Oral HIV Self-testing (oral HIVST) through Community-based screening (HIV CBS) in addition to other testing modalities aimed at hard-to-reach KPs, but the implementation cost is still not analysed. This study provides the cost and scale up cost estimation of HIV CBS in Jakarta and Bali, Indonesia.

**Methods:**

We estimated the societal cost of HIV CBS that was implemented through NGOs. The HIV CBS’s total and unit cost were estimated from HIV CBS outcome, health care system cost and client costs. Cost data were presented by input, KPs and areas. Health care system cost inputs were categorized into capital and recurrent cost both in start-up and implementation phases. Client costs were categorized as direct medical, direct non-medical cost and indirect costs. Sensitivity and scenario analyses for scale up were performed.

**Results:**

In total, 5350 and 1401 oral HIVST test kits were distributed for HIV CBS in Jakarta and Bali, respectively. Average total client cost for HIV CBS Self testing process ranged from US$1.9 to US$12.2 for 1 day and US$2.02 to US$33.61 for 2 days process. Average total client cost for HIV CBS confirmation test ranged from US$2.83 to US$18.01. From Societal Perspective, the cost per HIVST kit distributed were US$98.59 and US$40.37 for FSW and MSM in Jakarta andUS$35.26 and US$43.31 for FSW and MSM in Bali.

**Conclusions:**

CBS using oral HIVST approach varied widely along with characteristics of HIV CBS volume and cost. HIV CBS was most costly among FSW in Jakarta, attributed to the low HIV CBS volume, high personnel salary cost and client cost. Future approaches to minimize cost and/or maximize testing coverage could include unpaid community led distribution to reach end-users, integrating HIVST into routine clinical services via direct or secondary distribution and using social media network.

**Supplementary Information:**

The online version contains supplementary material available at 10.1186/s12913-024-10577-0.

## Background

Further decreases of AIDS-related deaths and HIV transmission can be achieved by identifying PLHIV who are unaware of their HIV status, early diagnosis and engagement with antiretroviral therapy (ART). By 2030, UNAIDS has established worldwide goals by 95% people living with HIV (PLWH) to know their status, of people who are known to be HIV-positive to be on ART, and of people who are currently receiving antiretroviral medication (ART) to have their viral load suppressed [1]. Based on this target, while the proportion of individuals who know their HIV status in Indonesia has shown an increase from 51% in 2019 [2] to 66% in 2021 [3], the proportion of people taking ART who are known HIV-positive decreased from 33% in 2019 [2] to 26% in 2021 [3]. These achievements remains far below the first and second 95% of UNAIDS target. These disparities were much lower in hard to reach KP particularly FSW and MSM. Among FSW, PLHIV who know their status and on ART among who know their status were 41% and 87% respectively. While among MSM, PLHIV who know their status and on ART among who know their status were 15% and 94% respectively [4, 5]. These KP contribute to the low achievement of UNAIDS first 95%. Therefore, the need to intensify HIV testing remains a critical step to surpass the Joint United Nations Programme on HIV/AIDS (UNAIDS) target to diagnose 95% of PLHIV by 2030. One of the strategy to increase HIV testing is by using innovative method HIV self-testing (HIVST). HIV self-testing (HIVST) is a process in which a person collects his/her own specimen (oral fluid or blood), and then performs HIV test using a HIV rapid diagnostic test, often in private setting, and interprets the result themselves either alone or assisted by someone they trusted [6]. HIVST is an innovative method shown by recent studies to be acceptable, feasible and effective in maximizing HIV testing uptake as a complement of conventional facility-based HIV testing [7–10]. The HIVST has the potential to expand diagnosis coverage in KPs not reached by facility-based testing and to minimize some of the socio-structural barriers to testing that currently exist. Previous studies found that barriers related to conventional facility-based HIV testing include social stigma, discrimination and geographic distance [11, 12]. These barrier also reported in a study conducted in Indonesia such as ongoing discrimination, social stigma, no privacy and no timely HIV testing [13]. HIVST allows people to self-test under conditions of privacy avoiding stigma and discrimination, increases repeat and partner testing, and, with appropriate dissemination strategies, may also reduce transportation barrier [14, 15].

To surpass the 95% UNAIDS target, Indonesia government since 2017 has develop an innovative HIV testing service (HTS), using HIV community-based screening (HIV CBS), to complement facility-based testing offered at healthcare facilities and mobile VCT offered through mobile clinic; through Oral HIVST approach. HIV CBS using oral HIVST is provided to target population who don’t want to have HIV test in health care facility/mobile clinic and prefer to conduct by themselves or assisted by peer leaders. In this HIV CBS programme, peer leaders have important role to educate clients about oral HIVST and give choices to client whether they choose to do oral HIVST independently or assisted by peer leaders. Since 2021, this HIV CBS using Oral HIVST approach has been nationally implemented started at 48 provinces in Indonesia including Jakarta and Bali (116 subdistricts for FSW and 130 subdistricts for MSM). Although the program implementation cost of Oral HIVST through HIV CBS may be higher (particularly after incorporating program or societal costs), impact studies demonstrate that there may be substantial public health advantages as it potentially increases diagnosis coverage, as such may offer value for money if implemented as a complement to current testing approaches [6].

In this study, we estimate the cost and scale up cost of HIV CBS implementation in 2 provinces in Indonesia (Jakarta and Bali) for FSW and MSM to guide project national scale-up and inform the sustainability of this HIV testing modality. The result of this study also contribute in minimizing the HIV prevention gap, as a part of the broader HIV treatment and prevention national plan in Indonesia.

## Methods

### Overview

We aim to measure the total cost of the HIV community-based screening (HIV CBS) intervention and also the average cost of HIV CBS per client in 2022 US$. We stratified the results based on type of key populations (FSW and MSM) and area (Jakarta and Bali). We estimated the cost of HIV CBS from the societal perspective (cost from health care system perspective and client perspective) by using micro-costing. We also provide one-way sensitivity analysis and scale up scenario analysis.

#### Study setting

The HIV community-based screening (HIV CBS) program was implemented in 2021–2022, integrated as part of the overall HIV prevention program. In the implementation of HIV CBS in Indonesia, OraQuick® is used as HIVST kits (further we use term oral HIVST), which were received from STAR III program. The distribution of the oral HIVST test kits to KPs at districts was coordinated through implementing organizations from national to district level. These implementing organizations also conduct key activities in support to HIV CBS implementation. Nationally, HIV CBS were conducted in 48 provinces (130 districts for FSW and 116 district for MSM). In Jakarta there are 5 districts while in Bali there are 3 districts that were counted as area with KPs. The overview of implementing organizations and HIV CBS locations are presented in Table 1. The HIV CBS were started since beginning of 2021 until November 2022.

**Table 1:** Overview implementing organization in Jakarta and Bali, Indonesia for HIV CBS program among FSW and MSM

Location	Jakarta	Bali
Number of districts covered	5 (District of Central Jakarta, West Jakarta, East Jakarta, North Jakarta, South Jakarta)	3 (District of Denpasar, Badung and Buleleng)
NGO^a^		
National (PR1, PR1, EPIC)	3 (PR1, PR2, EPIC)	2 (PR1, PR2)
Sub national (SR)	2 (FSW: SR1, MSM: SR2)	2 (FSW: SR3, MSM: SR4)
Local (SSR)	6 (FSW: SSR1, SSR2, SSR3, SSR4, SSR5; MSM: SSR6)	3 (FSW: SSR7, SSR8; MSM: SSR9)
Distribution channel	MSM, FSW	MSM, FSW
Number of trained peer leaders	78	21

^a^ Two NGOs known as Principal Recipients of International Donor Global Fund (PR 1 and PR 2) and 1 Project namely EPIC which was funded by FHI360 program; act as implementing organization at national level. Along with oral HIVST test kits distribution, these implementing organizations also conduct key activities (most of the HIV CBS-related key activities were integrated to the other HIV prevention program activities) from planning, training and HIV CBS manual development, stakeholder sensitization and engagement, socialization to KPs and peer leaders for monitoring and supervision. These key activities to support the HIV CBS including oral HIVST test kits distribution involved not only national PR but also – for the GF funding- NGOs at the sub national level (Sub recipient/SR); and districts level (Sub sub recipient/SSR). Specifically, a proportion of the HIV CBS among FSW were conducted under coordination of PR1 and its SR and SSR; and some other proportion by PR3, the same as among MSM, a proportion of the activities were under coordination of PR2 (and its SR and SSR) and also PR3

Among FSW and MSM, both did not start HIV CBS phases at the same time and oral HIVST kits mainly distributed directly through outreach activity at the community by peer leaders of district NGO (SSR). Peer leaders need to get the oral HIVST kits at the SSR office and then meet clients at agreed place and time. Client will be informed about the HIV CBS program and how to do the oral HIVST and sign informed consent form. Client may do oral HIVST with or without accompany of peer leaders. Based on Indonesian MoH technical guideline of HIV CBS [16], if the result of oral HIVST reactive, client need to be referred to health care facility to get HIV confirmatory test and proceed to ARV initiation if the test comeback positive.

#### Cost estimation

We estimated the cost of HIV CBS from the societal perspective (cost from health care system perspective and client perspective). The estimation of HIV CBS cost from health care system perspective followed the method described in the WHO training manual [17] and the Joint United Nations Program on HIV/AIDS (UNAIDS) [18]. The time frame of data collected was between early 2021 until end 2022. Costs data were collected from NGO as implementing organizations either through interview or details of financial expenditures report. Due to most of the HIV CBS-related key activities were integrated into other HIV prevention program activities, nearly no specific budget line contributed to HIV CBS program. Thus, if specific HIV CBS activities cost cannot be determined due to integration to other HIV prevention program, we assumed that this program contributed to some share of the related expenditures. These shares were determined by several assumptions. Details cost estimation methods from health care system perspectives, cost category, type and assumptions are presented and explained in Appendix 1 (including table A1 and table A2).

To measure the cost from client perspective, we collected data retrospectively through interviews using electronic semi structured questionnaire (Supplementary materials, appendix 3). The questionnaire was constructed based on previous studies that include the analysis of cost from patients’ perspective [19–24]. When developing the questionnaire, we carefully adapt the question and several steps was done to ensure the content validity. Respondents were reached from KPs who live in district in Jakarta and Bali Province given the following inclusion criteria: (1). Is FSW or MSM; (2). Live in Jakarta or Bali; (3). Never diagnosed as HIV positive; (4). Agree to join the study; and (5). Was tested for OFT in the last 1 month, which proven by consent form and OFT result. The exclusion criteria include: (1) No evidence of oral HIVST test and result. We interviewed 149 respondents conveniently. To estimate the minimal sample size, we used the formula for one sample mean in descriptive study with continuous variable as the outcome [25]. We utilized parameters (to measure standard deviation) previous published data on cost of HIVST conducted in Malawi, Africa [26] as no similar study has been conducted in Indonesia. Using these parameters, we found that the minimal sample size is 37. As we conducted the study in 2 key populations (FSW and MSM) and in 2 areas (Jakarta and Bali), we multiply this number with 4 and the sample size become 148. Thus, our sample size already fulfilled the minimal sample size for this study. Furthermore, several previous similar studies have shown that 50 patients would be sufficient to show the variation of the costs from patient perspective in one setting [21, 22]. Details of client cost’s data collection and cost inputs acquired to represent client cost were presented in Appendix 1 and table A1.

#### Outcome estimation

We collected outcome data from implementing organizations which are include: target of HIV CBS, number of oral HIVST test kits distributed/received by NGOs, number of clients who did HIV CBS, number of clients who did HIV CBS and the result were positive, number of clients who perform a confirmatory test of total screened positive, number of clients who result of confirmatory test come back positive and number of clients who initiate ART from total positive confirmation. This outcome was measured for those who went to HIV CBS through community outreach between January 2022 until December 2022.

#### Data analysis

We estimated the number of HIV CBS kits received by implementing organizations (NGOs) and the number of clients who did HIV CBS. We then divided each of these numbers to HIV CBS target to estimate uptake percentage. The total cost of the CBS HIV intervention is the sum of the total costs from the health care system and the client's perspective. We calculated the total health care system cost of HIV CBS intervention and its categories (by phase were categorized into start-up and implementation phase, and for each phase was categorized into capital and recurrent cost). To enable identifying the cost driver, for each capital and recurrent cost, we also calculated the cost component (for example: in capital cost includes equipment, in recurrent cost include salaries and office running cost). For the total client cost, we multiply the average client cost to total number of clients who did HIV CBS. We calculate average client cost by summing average direct medical, average direct non-medical and average indirect cost per clients stratified by type of KPs and province.

Based on the total HIV CBS cost, we estimated the average cost of HIV CBS, per client who did HIV CBS. We assumed that all costs associated with the HIV CBS were in addition to the costs of standard-of care HIV testing services. Cost and outcome data were also presented based on KPs and region. Cost data were annualized using a 3% discount rate, which is common rate that utilized for economic evaluation of health care programme [27–29]. Capital cost were also annualized considering the useful life of the capital item for depreciation purpose and discount rate, to obtain the value of capital for one time period by dividing the replacement cost of capital by the annualization factor [30]. All costs were estimated in 2022 USD dollars using average annual exchange rates. All analyses were conducted in Excel Version 15.19.1. The formula for this cost analysis is presented here:

$$Total\;HIV\;CBS\;Cost\;=\;Total\;HIV\;CBS\;cost\;(health\;care\;system)\;+\;Total\;HIV\;CBS\;cost\;(client)$$

$$The\;average\;cost\;of\;HIV\;CBS=Total\;HIV\;CBS\;cost\;/Number\;of\;Client\;who\;did\;HIV\;CBS$$

Where:

1. Total HIV CBS Cost were the total intervention cost of HIV CBS from health care system perspective and also from client perspective
2. Total HIV CBS health care system cost = Total startup phase cost + Total implementation phase cost
3. Total HIV CBS client cost = Average client cost multiply by number of clients who did HIV CBS

#### Sensitivity analysis

We conducted a one-way sensitivity analyses, using tornado diagrams, to assess the impact of key cost assumptions on the average cost per oral HIVST test kits distributed to client for screening (HIV CBS). We varied the discount rate used to annualized costs to 0 and 13% (base case is 3%) to capture the impact of not discounting. We varied annualization (economic life years) time frames: training & sensitization were varied between 1 and 3 years (base: 2 years) and equipment life between 2.5 and 7.5 years (base: 5 years) to assess the impact of the assumed project life years on costs. We also varied the cost which shown as key drivers of total cost by ± 10% from the base case in which these include: personnel salaries, cost of supervision, coordination meeting at SR level and office running cost.

#### Scenario analysis for scale up

Expecting the transition of the HIV CBS program is taking step by step process integrated to ministry of health program, we set 4 scenarios that corresponded to 5 years of scale up program and cost variation (Table 2). To assess the impact of varying cost input in national and subnational level, we reduced the national level cost incurred by PR and subnational level incurred by SR by 20% to 80% for 5 years (capital cost during startup and implementation phase which included sensitization and training, supervision and monitoring, salaries), thus showing effort to minimize dependency to international donor funding. At local level, we reduced the personnel salary for peer leader and program management by 20% to 80% for 5 years hoping that KP will self-manage to do oral HIVST without the need to do outreach activity conducted by peer leaders. We scale up the program by increasing the number of oral HIVST test kits distributed to client for screening by percentage of targeted OFT (50%-90% for 5 years). All these variations were in 5 scenario which each proceed simultaneously for all inputs. Finally, we estimated a best- and worst-case scenario, the point where all the parameters yield the lowest/highest unit cost per kit distributed for screening.

**Table 2:** Selected parameters for the scenario analysis of costs at scale-up in Jakarta and Bali, Indonesia

	**Scenario 1**	**Scenario 2**	**Scenario 3**	**Scale up**
	**National implementing partner cost (PR) (% reduction of 2022 capital cost (activity cost) during startup and implementation)**	**Sub-national implementing partner cost (SR) (% reduction of 2022 capital cost (activity cost) during startup and implementation; and personnel salaries)**	**Local implementing partner costs (% reduction of 2022 capital cost (activity cost) during startup and implementation; and personnel salaries)**	**Reaching oral HIVST kits distribution target for HIV CBS (% of target)**
**2022**	Current state	Current state	Current state	Current state
**2023**	-20%	-20%	-20%	50%
**2024**	-40%	-40%	-40%	60%
**2025**	-50%	-50%	-50%	70%
**2026**	-60%	-60%	-60%	80%
**2027**	-80%	-80%	-80%	90%

## Results

### HIV CBS outcome

**Fig. 1:**
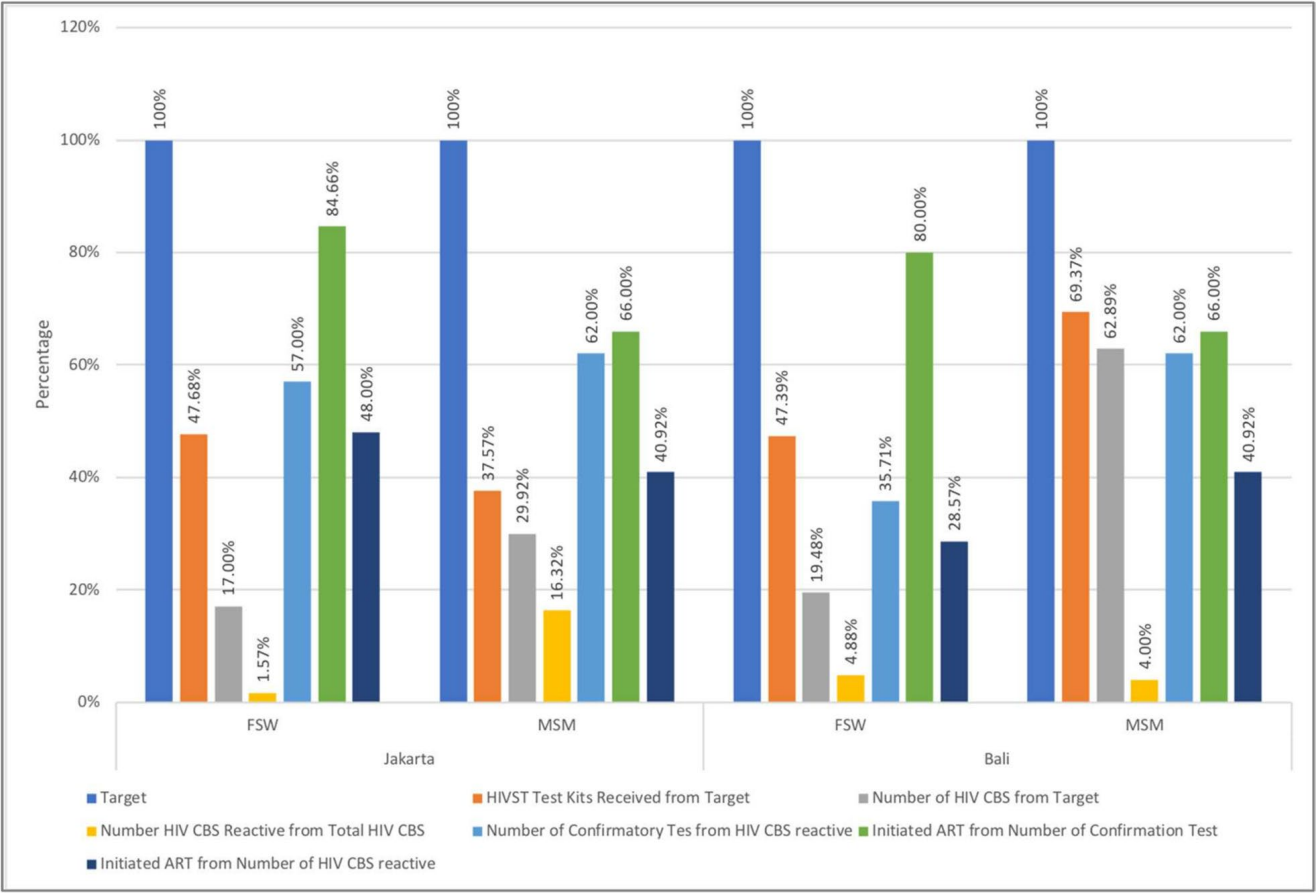
Outcome cascade of care (in percentage). Outcome data includes oral HIVST test kits received, HIV CBS, HIV CBS reactive, confirmation test, ART initiation

During HIV CBS implementation costing periode (January 2022 to Desember 2022) approximately a total of 5350 (41.82% of target) and 1401 (56.35% of target) oral HIVST kits were received by implementing partner organization in Jakarta and Bali; 3134 (24.49% of target) and 924 (37.17%) were distributed to client for screening (HIV CBS) in Jakarta and Bali against the approximate target of 12793 and 2486 among KP through a total of 78 and 21 peer leaders (PL) in Jakarta and Bali, respectively. These coverages of HIV CBS achieved was still around 10% of the HIV CBS national achievement figure (average: 31.17%, ranged: 20% to 42%). Of all oral HIVST test kits distributed to clients, 17.54% were distributed to FSW and 33.88% distributed to MSM for HIV screening. In Jakarta, HIVST kits distributed to FSW for screening were 17.00% and for MSM were 29.92% from target. While in Bali 19.48% were distributed to FSW and 62.89% distributed to MSM from target. Overall HIV CBS screening positivity rate was 10.25% for Indonesia across key populations. In Jakarta and Bali, HIV CBS positivity ranged between 2.3% among FSW and 13.58% among MSM. Based on location, HIV CBS positivity ranged between 12.03% in Jakarta and 4.27% in Bali. The proportion of recipients screening positive with an oral HIVST kit who reported to have obtained confirmatory testing ranged from 46.42% in FSW to 62.11% among MSM. Out of those clients with reactive HIV CBS who obtained confirmatory testing, between 66.87% reported to have initiated ART. This means that out of everyone screening positive, approximately 40.75% initiated ART. Details of care cascade are presented in Fig. 1.

### Cost from client perspective

A total of 149 participants from MSM and FSW in Jakarta and Bali who did HIV CBS were recruited. Details of client’s characteristics and average client’s cost is described in Appendix 2, table A3, table A4. To estimate total of HIV CBS intervention cost from client perspective, we multiply the average of client cost who did HIV CBS to number of clients who did HIV CBS. By applying this result to the total number of clients who did HIV CBS, the total HIV CBS cost from client s perspective were US$45,905.62. When we incorporate the number of clients among MSM and FSW in Jakarta and Bali to measure the unit cost, we found that the cost per oral HIVST kit distributed for HIV CBS from client’s perspective were $18.86 and $11.94 for FSW and MSM in Jakarta, while in Bali were $3.76 and $2.14 for FSW and MSM in Bali.

### Cost from health care system and societal perspective

From the health care system perspective, the total intervention cost of HIV CBS in Jakarta and Bali were US$174,753.82 (accounted for 79.2% of societal cost). Health care system cost in Jakarta accounted for 79.8% (US$139,472.44) and in Bali account for 20.2% (US$35,281.38). In Jakarta, the HIV CBS intervention costs were calculated as US$73,060.15 and US$66,412.29 for FSW and MSM respectively (or 52.4% and 47.6% from total health care system cost in Jakarta); while in Bali the total HIV CBS intervention costs were calculated as US$9,042.16 and US$26,239.22 for FSW and MSM (or 25.6% and 74.4% from total health care system cost in Bali) respectively (Table 3). From the health care system perspective, intervention cost of HIV CBS per oral HIVST kit distributed to clients for HIV CBS were $80.02 and $29.90 for FSW and MSM in Jakarta, while in Bali were $31.51 and $41.18 for FSW and MSM in Bali.

**Table 3:** HIV CBS per client screened cost breakdown and key cost contributors (in 2022 US$)

Input	Jakarta				Bali			
	FSW		MSM		FSW		MSM	
	Cost	%	Cost	%	Cost	%	Cost	%
Health care system cost								
Start up								
Capital cost								
Building and Spaces	$261.48	0.3%	$140.71	0.2%	$56.09	0.6%	$131.53	0.5%
Equipment								
Furniture	$93.52	0.1%	$25.11	0.0%	$60.02	0.6%	$83.40	0.3%
Hardware	$127.33	0.1%	$181.12	0.2%	$42.00	0.4%	$54.25	0.2%
Vehicle	$34.63	0.0%	$8.41	0.0%	$23.19	0.2%	$32.36	0.1%
Multimedia	$3.24	0.0%	$2.27	0.0%	$0.54	0.0%	$0.54	0.0%
Storage equipment	$4.77	0.0%	$9.80	0.0%	$3.27	0.0%	$2.91	0.0%
Total equipment cost	$263.49	0.3%	$226.71	0.3%	$129.02	1.3%	$173.47	0.6%
Sensitization (coordination, socialization, technical assistance), guideline development and trainings activity	$1,426.33	1.6%	$6,810.63	7.6%	$400.06	4.0%	$5,534.78	20.1%
Total capital costs	$1,951.31	2.2%	$7,178.05	8.0%	$585.17	5.8%	$5,839.79	21.2%
Total start-up cost	$1,951.31	2.2%	$7,178.05	8.0%	$585.17	5.8%	$5,839.79	21.2%
Implementation								
Capital cost								
Building and Spaces	$42.52	0.0%	$20.09	0.0%	$21.26	0.2%	$33.48	0.1%
Supervision, monitoring, stakeholder meeting and internal coordination	$3,762.07	4.2%	$1,259.94	1.4%	$1,864.85	18.4%	$1,143.91	4.1%
Total capital costs	$3,804.59	4.2%	$1,280.03	1.4%	$1,886.11	18.6%	$1,177.39	4.3%
Recurrent cost								
Personel								
Salaries national level	$2,732.66	3.0%	$8,632.79	9.6%	$231.56	2.3%	$52.24	0.2%
Salaries sub-national level	$188.46	0.2%	$313.01	0.3%	$136.90	1.4%	$356.56	1.3%
Salaries of peer leaders	$44,702.24	49.7%	$32,266.68	36.0%	$1,882.27	18.6%	$13,404.88	48.6%
Salaries local level: program management	$6,523.57	7.2%	$2,967.50	3.3%	$459.62	4.5%	$1,610.76	5.8%
Total personnel cost	$54,146.94	60.2%	$44,179.97	49.3%	$2,710.34	26.8%	$15,424.44	55.9%
Supplies								
Packing, handling and delivery	$658.35	0.7%	$1,158.72	1.3%	$144.36	1.4%	$486.30	1.8%
Office running cost	$3,318.92	3.7%	$3,643.47	4.1%	$1,134.62	11.2%	$805.51	2.9%
Personal Protective Equipment (PPE) and Storage Kit for Outreach Workder	$1,554.67	1.7%	$238.21	0.3%	$503.27	5.0%	$222.62	0.8%
OFT test kits	$7,625.37	8.5%	$8,304.24	8.9%	$2,078.29	20.5%	$2,093.18	7.6%
Social media campaign and hosting	$0.00	0.0%	$429.58	0.5%	$0.00	0.0%	$189.99	0.7%
Total supplies cost	$13,157.32	14.6%	$13,774.22	14.8%	$3,860.54	38.1%	$3,797.60	13.8%
Total recurrent cost	$67,304.25	74.8%	$57,954.20	62.4%	$6,570.88	64.9%	$19,222.04	69.6%
Total implementation cost	$71,108.85	79.0%	$59,234.23	63.8%	$8,456.99	83.6%	$20,399.43	73.9%
Total health care system cost	$73,060.15	81.2%	$66,412.29	71.5%	$9,042.16	89.3%	$26,239.22	95.1%
Client cost								
Screening								
Direct non medical costs	$11,137.34	12.4%	$16,148.89	18.0%	$35.93	0.4%	$630.98	2.3%
Indirect costs	$5,628.76	6.3%	$6,117.69	6.8%	$946.70	9.4%	$616.68	2.2%
Confirmatory test								
Direct medical costs	$70.98	0.1%	$1,656.94	1.8%	$65.49	0.6%	$86.08	0.3%
Direct non medical costs	$81.82	0.1%	$1,663.69	1.9%	$8.08	0.1%	$7.05	0.0%
Indirect costs	$29.14	0.0%	$910.42	1.0%	$22.25	0.2%	$20.16	0.1%
Total Client Cost	$16,948.04	18.8%	$26,497.64	29.6%	$1,078.46	10.7%	$1,360.95	4.9%
Total HIV CBS Cost	$90,008.20	100.0%	$92,909.92	100.0%	$10,120.62	100.0%	$27,600.17	100.0%
Cost per HIVST distributed for screening (HIV CBS) (health care system perspective)	$80.02		$29.90		$31.51		$41.18	
Cost per HIVST distributed for screening (HIV CBS( (client perspective)	$18.56		$11.07		$3.76		$2.14	
Cost per HIVST distributed for screening (HIV CBS) (societal perspective)	$98.59		$40.37		$35.26		$43.31	

From societal perspective, the total cost of HIV CBS in Jakarta and Bali were US$220,638.91 where in Jakarta accounted for 82.9% (US$182,918.12) and in Bali account for 17.1% (US$37,720.79). In Jakarta, the HIV CBS societal costs were calculated as US$90,008.20 and US$92,909.92 for FSW and MSM respectively (or 49.2% and 50.8% from total societal cost in Jakarta); while in Bali the total HIV CBS societal costs were calculated as US$10,120.62 and US$27,600.17 for FSW and MSM (or 26.8% and 73.2% from total societal cost in Bali) respectively. From societal perspective the cost per oral HIVST kit distributed for HIV CBS were $98.59 and $41.83 for FSW and MSM in Jakarta, while in Bali were $35.26 and $43.31 for FSW and MSM in Bali.

### Sensitivity analysis

**Fig. 2:**
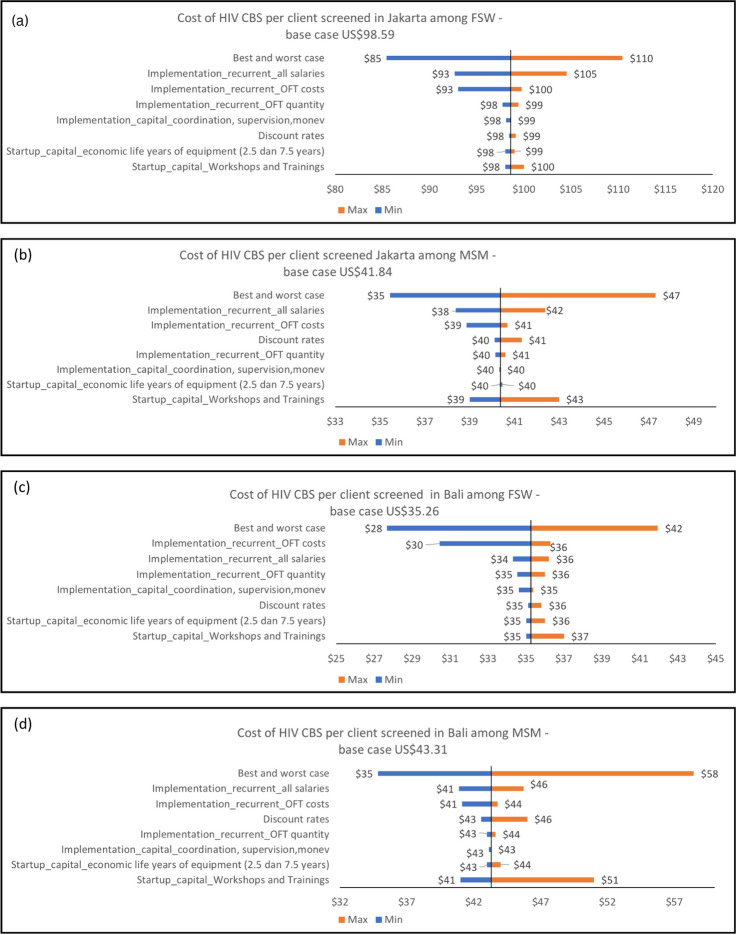
**a**-**d** Tornado diagram from deterministic sensitivity analysis (univariate analysis). Variation include the discount rate used to annualized costs to 0 and 13% (base case is 3%) to capture the impact of not discounting. Annualization (economic life years) time frames: training & sensitization were varied between 1 and 3 years (base: 2 years) and equipment life between 2.5 and 7.5 years (base: 5 years) to assess the impact of the assumed project life years on costs. Cost which shown as key drivers of total cost by ± 10% from the base case (personnel salaries, cost of supervision, coordination meeting at SR level and office running cost) were also varied

Figure 2 show results from the univariate sensitivity analyses by KPs and province. Overall, our unit costs per HIV CBS to client screened remained robust when key cost parameters were varied to minimum or maximum with maximum changed were still around 10% of the base case. Moderate effect (more than around 15–20% change) to base case shown among FSW in Bali when OFT purchasing cost set to minimum (base case decreased by 13.6%) and among MSM in Bali when economic life years of building and equipment set to 7.5 years (the average cost were increase by 18.62%). Strong effect was shown when all key parameters set to minimum and maximum, the average cost of HIV CBS variation show a decrease by 21.5% to an increase by 34.98%. Across all area and key populations, varying salaries in all personnel level resulted in slight effect to cost per oral HIVST distributed (around 2.5% to 5% from base case). The same also happen if varying coordination, supervision and monitoring costs at all area and KP which resulted to slight effect on base case.

### Scenario analysis for scale up

**Fig. 3:**
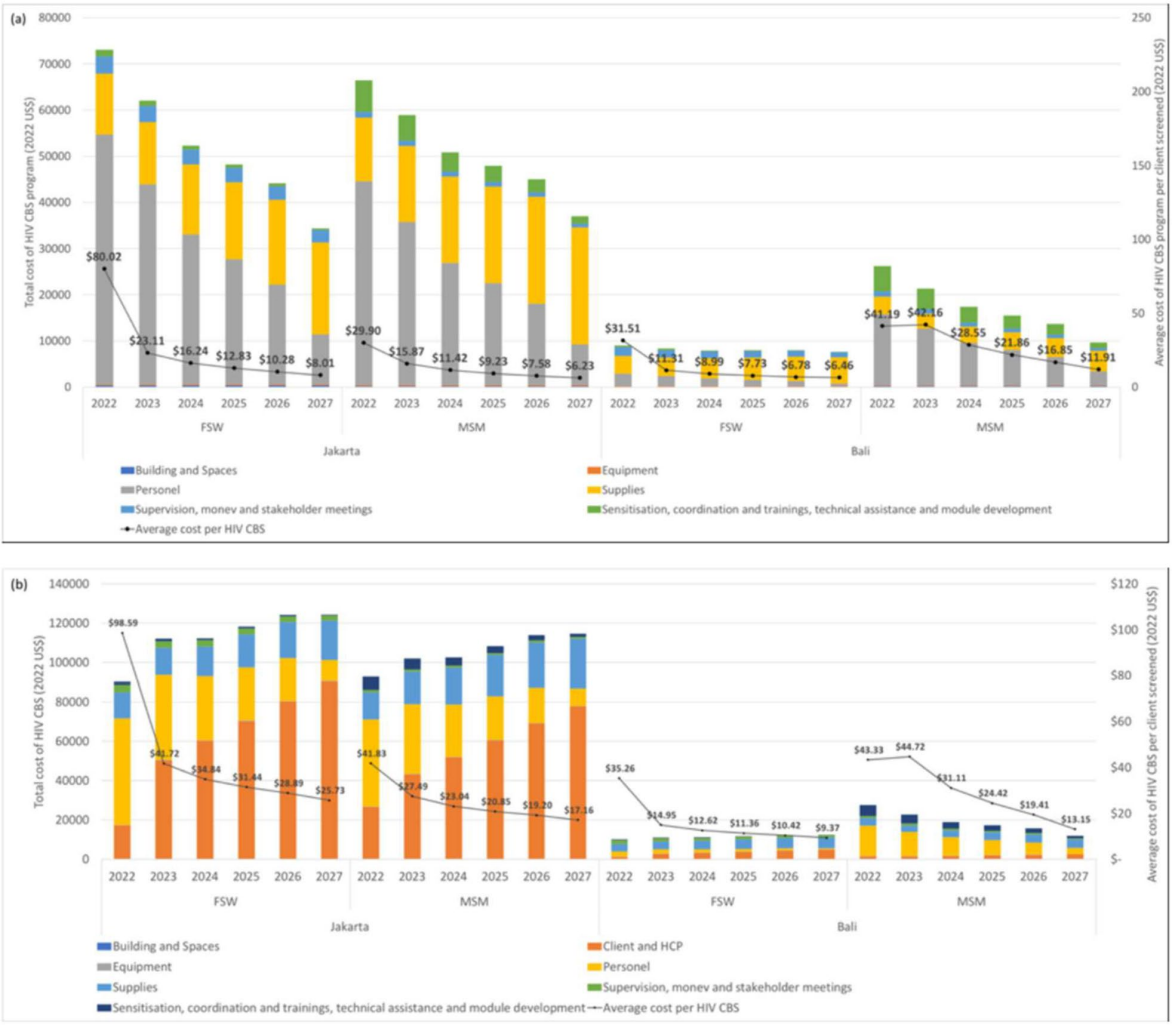
**a**-**b** Total and average HIV CBS cost per client from health care system (**a**) and societal perspective in current situation (2022) and scale-up (2023–2027) by location and key population. To assess the impact of varying cost input in national and subnational level, we reduced the national level cost incurred by PR and subnational level incurred by SR by 20% to 80% for 5 years (capital cost during startup and implementation phase which included sensitization and training, supervision and monitoring, salaries), thus showing effort to minimize dependency to international donor funding. At local level, we reduced the personnel salary for peer leader and program management by 20% to 80% for 5 years hoping that KP will self-manage to do oral HIVST without the need to do outreach activity conducted by peer leaders. We scale up the program by increasing the number of oral HIVST test kits distributed to client for screening by percentage of targeted OFT (50%-90% for 5 years). All these variations were in 5 scenario which each proceed simultaneously for all inputs. Finally, we estimated a best- and worst-case scenario, the point where all the parameters yield the lowest/highest unit cost per kit distributed for screening

Costs at scale-up for scenario are presented by area and key group in Fig. 3. Across areas, years, and key groups, the trend is an overall decrease in total costs and unit costs. Although we estimate variation between area and key groups, overall cost drivers from health care system perspective are recurrent cost including personnel salary and supplies (OFT quantity) and capital cost due to fix activities (training during startup phase and supervision and monitoring evaluation during implementation). When we incorporate societal cost (client and health service provider costs), the cost driver become client and health care provider cost followed by personnel of program implementor and OFT quantity. The unit cost from health care system and societal perspective were decreased as the scale up progresses from 2022 to 2027.

## Discussion

We have estimated the cost of implementing HIV Community Based Screening (HIV CBS) using Oral HIV Self testing (Oral HIVST) for KPs FSW and MSM in Jakarta and Bali, Indonesia. From societal perspective, we found that the average cost of HIV CBS ranged from as low as US$35.26 among FSW in Bali to as high as US$98.59 among FSW in Jakarta. The cost per oral HIVST distributed were lowest among FSW in Bali due to the number of OFT distributed to Bali among FSW were lowest compare to other area or other KP (10.3% from total OFT distributed) resulted in lowest HIV CBS program cost; and low client cost). The cost per oral HIVST distributed were highest among FSW in Jakarta may due to several factors. First, number of peer leaders who reached FSW in Jakarta were the largest but the number of client reached for HIV CBS were less that 25% of total HIV CBS in Jakarta and Bali, thus the program cost of HIV CBS among FSW in Jakarta were consequently larger than any other group or area since that the cost driver of health care system cost were personnel salary, and also activities with expenses related to number of person involved (training, supervision). Another reason may due to, from client cost, the non-medical cost of HIV CBS during screening and confirmatory phase among FSW in Jakarta were remarkably high. The high non-medical cost of client among FSW in Jakarta were may attributed to the high transportation cost (the transportation cost were 2–6 times higher in Jakarta compare to Bali). This is reasonable as Jakarta categorized as megapolitan city with high traffic congestion, thus the transportation charge and time is higher among other area [31]. Furthermore, FSW is hard to reach KP group even though live in the city, as they usually live in slump and poverty-identical area lacking easy structural access to health facility (limited transportation mode and geographical distance) [32].

Moreover, our finding regarding the average HIV CBS client cost leads to some important observations. First, we found our average costs were relatively higher compare to study conduct by Maheswaran et al. [33] even though the lowest average client cost of HIV CBS in our study were US$4.78 among MSM in Bali. While our study include cost estimates of outreach phase (to provide information), confirmatory test and communication cost; study by Maheswaran [33] only measures screening phase and did not measures direct non-medical cost nor indirect cost. Second, our finding is also higher compared to the study conducted by Verstraaten et al. (2017), who found that the non-health care cost of mobile VCT (who meet client at hot spot thus have similar setting with HIV CBS was US$0.44 (covert to US$ in 2022). However, study conducted by Verstraaten et al. did not estimate the outreach phase cost to provide HIV testing information and communication cost in their non-health care cost components.

To our knowledge, this is the first published study estimating cost of HIV self-testing in Indonesia or others similar settings in South East Asia, thus we cannot make study comparation to other location in Indonesia or South East Asia. The costs per oral HIVST kits distributed to client for screening in our study across all area and KPs are higher compare to other similar studies in African countries [33–37]. This finding may due to several factors. First, the number of HIV CBS estimated in this study were lower compare to other studies, which only 3134 (24.49% of target) and 924 (37.17% of target) distributed to client for screening in Jakarta and Bali against the approximate target of 12793 and 2486 among KP in Jakarta and Bali, respectively. This may be explained because many of other studies have oral HIVST volumes that were higher due to targeting the general population or the whole country, while our study only KP from 2 provinces. Another explanation is that other community-based HIVST studies that were reported have various HIVST delivery models [36, 37], while in our study, the delivery model were majorly from peer leader who reached the KP which initial screening were mostly conducted in 1 days (78.5%) but still contributed to high proportion of peer leaders cost to reach clients. On the other side, despite the lower HIVST volume distributed among MSM and FSW in Jakarta and Bali (didn’t reach target), the program cost both from health care system perspective and societal perspective were quite high despite this study were only in 2 provinces Jakarta and Bali.

The scale up scenario analysis suggests that along with the year progresses, HIVST can exhibit economies of scale. When comparing year 2022 with the other years to which scenario analysis were applied, we found variable scale economies between key populations and areas. We estimated average cost reduction of community based HIVST per year was about 67.01% (FSW) and 48.49% (MSM) from the observed societal cost in 2022 in Jakarta; whereas in Bali we found 65.01% (FSW) and 30.96% (MSM) cost reduction. From health care system cost, average cost reduction of community based HIVST per year was about 82.39% (FSW) and 66.33% (MSM) from the observed 2022 cost in Jakarta; whereas in Bali we found 73.80% (FSW) and 41.10% (MSM) cost reduction.

### Limitations

Our study has several limitations. First, the findings of our cost analyses are limited to unit cost per kit distributed to client for screening until client did confirmation test as described in technical guidance for HIV CBS. We did not include the cost for ART initiation as the ART initiation cost per year for client who HIV positive were quite high and the HIV positive proportion compare to number screened were low thus may lead to very high cost per HIVST distributed which make our result may not comparable to other study [35, 37]. Second, in our study, the oral HIVST distribution were conservative through peer leader outreach activity to key populations of FSW and MSM, restricting the number of kits distributed to larger scale, consequently, costs were likely higher than possible future routine implementation. Third, the client cost estimation could be affected by recall bias as we collected the cost data retrospectively. Forth, we did not account for invalid result of oral HIVST as this study is a cost evaluation of oral HIVST during program implementation of HIVCBS and no invalid result data were reported by the program implementer.

## Conclusions

Across key populations, the total and unit cost of HIV CBS using oral HIVST approach in Jakarta and Bali, Indonesia; varied widely along with characteristics of HIV CBS volume and cost. HIV CBS was least costly among FSW in Bali attributed to the low personnel cost and low client cost. HIV CBS was most costly among FSW in Jakarta attributed to the low HIV CBS volume, high personnel salary cost and client cost. In transition to scale-up where aiming to reduce cost from program implementor and peer leader and trying to increase oral HIVST distributed, this model shows large potential for substantial economies of scale as programs scale-up and mature. However, this need to be carefully considered whether it is possible to manage HIV CBS program without or with minimal involvement of NGOs and peer leaders as other variables need to be explored other than cost and outcome (such as resource management capacity, approach and relation to key populations). Especially when discussing about hard to reach populations (high prevention gap) in Indonesia such as MSM [38] and FSW, their sexual partner and client, which need to be carefully considered which strategy is more promising thus increasing HIV testing and closing the prevention gap [39]. Further distribution strategies need to be explored to ensure which strategies is more effective for several KPs. Every population risk for HIV have different characteristic, thus priority need to be put between trying to target KP with financial or other barriers to obtaining HIV testing in health services, that is people living in settings with high undiagnosed HIV or remote communities, and groups such as men and adolescents [40]. Alternative strategies to contain program cost is by integrating HIVST within community health service, secondary distribution and peer network approach or through online social media.

### Supplementary Information

**Additional file 1:** **Appendix 1.** Methods: Cost Estimation.

**Additional file 2:** **Appendix 2.** Cost from Client Perspective.

**Additional file 3:** **Appendix 3. **Questionnaire for client cost.

## Data Availability

The datasets used and/or analysed during the current study are available from the corresponding author on reasonable request.

## Abbreviations

ART: Antiretroviral Therapy
FSW: Female Sex Worker
HIV: Human Immunodeficiency Virus
HIVST: HIV Self-testing
HIV CBS: HIV Community-Based Screening
KP: Key Population
MSM: Men who have Sex with Men
OFT: Oral Fluid Test
PR: Principal Recipient
SR: Sub Recipient
SSR: Sub Sub Recipient
