# Supplementary material for: Costs and scale-up costs of community-based Oral HIV Self-Testing for female sex workers and men who have sex with men in Jakarta and Bali, Indonesia
Source: BMC Health Serv Res. 2024 Jan 22;24:114. doi: 10.1186/s12913-024-10577-0 (PMC10802071; doi:10.1186/s12913-024-10577-0)
Supplement: Supplementary file 1 — Additional file 1: Appendix 1. Methods: Cost Estimation. [file 12913_2024_10577_MOESM1_ESM.docx]

**Appendix 1. Methods: Cost Estimation**

***Cost from health care system perspective***

Costs data were collected from NGO as implementing organizations either through interview or details of financial expenditures report. These included startup and implementation phases costs. Each NGO and its KPs has different schedule in startup and implementation phase. Each phase included capital and recurrent costs input. Startup costs include cost of training, sensitization, socialization, equipment purchasing, website setup and social media campaign and HIVST test kits distribution costs. Implementation costs include cost of activity (supervision, monitoring and evaluation), monthly/annual office rent, office running costs, salary, and personal protective equipment (PPE). Based on the level of implementing organizations, costs also will be calculated from PR, SR and SSR level. Detail of activity type, input and data source for each category is presented in Table A1. During the implementation of HIV CBS in Indonesia, the HIVST cost was US$2.99 per test kits.

Due to most of the HIV CBS-related key activities were integrated into other HIV prevention program activities, nearly no specific budget line contributed to HIV CBS program. Thus, if specific HIV CBS activities cost cannot be determined due to integration to other HIV prevention program, we assumed that this program contributed to some share of the related expenditures. These shares were determined by several assumptions.

1. The first assumption was based on the share of target/activity of this program to the overall program target/activities which we acquired the information from NGO management staff. Based on the interview, HIV CBS through HIVST approach is part of HIV prevention program, which accounted for a share of all HIV prevention program. Thus, if cost of HIV CBS data is integrated with other HIV prevention program expenditure (not directly to HIV CBS spending), we multiply the cost data with the percentage of HIV CBS expenditure to overall expenditure. Details of cost category, type and assumptions
2. As we only measure the cost of HIV CBS in Jakarta and Bali, the financial data in SR and PR level were based on type of cost data. If the unit cost data is based on the location of the activity, we used the proportion of districts in Jakarta or Bali to all districts in Indonesia. On the other hand, if the unit cost is number of persons, we used the proportion of number of PL in Jakarta or Bali compared to Indonesia. Detail of this share is presented in Table A2.

*Client Cost*

To measure the cost from client perspective, we collected data retrospectively through interviews using electronic semi structured questionnaire. The questionnaire was constructed based on previous studies (19-124) that include the analysis of cost from patients’ perspective. When developing the questionnaire, we carefully adapt the question and several steps was done to ensure the content validity. We make sure the question constructed base on costing method theory (including the type of variables) and also involving team with expertise on costing method and HIV). The previous questionnaire that we use also has undergone several revisions and the one that we used has been tailored to suit our study. As our questions are straightforward (e.g. socio-economic, cost spent, time spent), we did not test the validity of our questionnaire. We also conducted group workshop for peer leader who helped in client interview to ensure the questionnaire reflect the real HIV CBS in the field. Moreover, the questionnaire is not self-filling, thus enumerator asked and explained the questionnaire directly to the respondents.

To reduce the inherent weakness of retrospective data, we have tried to do several strategies. First, even though one of the inclusion criteria is client who have undergone oral HIVST, but we ensure that peer leaders reached the client that just recently did HIVST (preferably less than 1 month). This to make sure the time of test to interview is not too long as the more recent event are easier to recall . Second, prior to interview, during the preparation phase, we conduct workshop for peer leaders to inform about the interview questionnaire, then the questionnaire was pilot tested and revised to ensure questions were clear and precise. This was done to reduce variation in comprehension and allowed practice and evaluation of interviewer technique to ensure consistency across interviews. Third, the questions in the questionnaire were created sequentially based on the stages of HIV CBS (information delivery – screening/self-testing – confirmatory test), aiming for memory aid. The last, we use face to face interview as mode of data collection and the interview was conducted in preferred client space, aiming for ease of concentration and memory aid.

The cost of HIV CBS from client perspective were included starting from pre-test (information delivery), HIVST process to confirmatory test. Of all these 3 phases, respondents were asked about direct non-medical and indirect costs that they or accompanying family member or care takers incurred in accessing HIV CBS services. Direct non-medical costs included the cost of transportation, food and drinks whilst waiting, and other costs incurred as a consequence of testing (internet, parking). Indirect costs included time off work multiplied by self-reported expenditure. In addition, we recorded the total time spent testing, including travel and waiting time. In the phase of confirmatory test, we also asked direct medical cost such as administration fee, laboratories fee and service fee. Details of cost inputs acquired to represent client cost were presented in Table A1.

**Table A1. Structure of activities, inputs and data source**

| **Activities** | **Cost input** | **Resource** |
| --- | --- | --- |
| **Health care system cost** | | |
| *Start up: capital* | |  |
| Equipment | Furniture, hardware , vehicle, multimedia,storage equipment | Staf interview, expense report |
| Sensitization (coordination, socialization, technical assistance), guideline development and trainings activity | Mobile internet, stationary, honorarium, meeting package/accommodation, per diem and/or transportation fee, meal allowance. | Staf interview, expense report |
| *Implemention: capital* | | |
| Building and Spaces | Warehouse storage, fffice rental | Staf interview, expense report |
| Supervision, monitoring, stakeholder meeting and internal coordination | Mobile internet, stationary, honorarium, meeting package/accommodation, per diem and/or transportation fee, meal allowance. | Staf interview, expense report |
| *Implementation: recurrent* | |  |
| Personel (Salary at national, sub-national and local level) | Basic salaries, 13-th salarries, health insurance, social insurance | Staf interview, expense report |
| Supplies | |  |
| Packing, handling and delivery | Handling fee, shipping fee, repacking fee, VAT 10%, Cardboard 40x25x25 | Staf interview, expense report |
| Office running cost (ORC) | Water, stationery, vehicle gasoline, bank admin fees, cleaning service, photocopying, internet, security, pool, electricity, logistics, purchase of checks, trash, ac service, printer ink, office telephone, gardener | Staf interview, expense report |
| Personal Protective Equipment (PPE) and Storage Kit for Outreach Worker | Thermapack insulated mailers , bag, emergency lamp/Flash Light, face mask, hand glove, handsanitizer, Ice gel, Map | Staf interview, expense report |
| OFT test kits | OFT quantity | Staf interview, expense report |
| Social media campaign and hosting | Facebook ads, internet hosting, Social media campaign | Staf interview, expense report |
| **Client cost** |  |  |
| *Non-health care cost* | | |
| Information delivery   - Direct non-medical cost - Indirect cost (productivity lost) | Direct non-medical cost: transportation, food and drinks whilst waiting, and other costs incurred as a consequence of testing (internet, parking)  Indirect costs included time off work multiplied by self-reported expenditure. In addition, we recorded the total time spent testing, including travel and waiting time | Client interview |
| Screening (HIVST)   - Direct non-medical cost - Indirect cost (productivity lost) |  | Client interview |
| Confirmation test   - Direct non-medical cost - Indirect cost (productivity lost) |  | Client interview |
| *Health care cost* | | |
| Confirmation test | Direct medical cost: administration fee, laboratories fee and service fee | Client interview |

**Table A2. Assumptions on expenditure allocation of HIV CBS implementation**

| **Expenditure items** | **Expenditure’s allocation assumptions** | | | | | | |
| --- | --- | --- | --- | --- | --- | --- | --- |
|  | **1^st^ assumption** | | **2^nd^ assumption** | | | | |
|  | **Proportion** | **Source** | **Proportion** | | | | **Source** |
|  |  |  | **Bali FSW** | **Bali MSM** | **Jakarta FSW** | **Jakarta MSM** |  |
| *National level* |  |  |  |  |  |  |  |
| Building (office rent) and equipment, activities (capital, recurrent), salary, ORC | 6% | Interview with NGO staff | 2% | 2% | 4% | 4% | Proportion of peer leaders/sub-districts number in Bali or Jakarta among total number of peer leaders/sub-districts |
| Building and equipment (warehouse storage) | 20% |  |  |  |  |  |  |
| Supplies_OFT | 100% | Expenditure data | - | - | - | - | - |
| *Sub-national* |  |  |  |  |  |  |  |
| Building (office rent) and equipment, activities (capital, recurrent), ORC | 11% | Interview with NGO staff | 5% | 15% | 7% | 13% | Proportion of peer leaders/sub-districts number in Bali or Jakarta among total number of peer leaders/sub-districts |
| Personel salary: monitoring and evaluation staf | 7% |  |  |  |  |  |  |
| Personel salary: program officer and coordinator | 9% |  |  |  |  |  |  |
|  |  |  |  |  |  |  |  |
| Personel salary: finance | 11% |  | 6% | 9% | 8% | 9% | Based on proportion of SSR number |
| *Local* |  |  |  |  |  |  |  |
| Building (office rent) and equipment, activities (capital, recurrent) | 11% | Interview with NGO staff | - | - | - | - | - |
| Personel salary: program management | 8% |  | - | - | - | - | - |
| Personel salary: peer leaders | 11% |  | - | - | - | - | - |
| Supplies_PPE | - |  | 20% | 19% | 17% | 30% | Based on HIV CBS achievement |
| Supplies_ORC | 11% |  | - | - | - | - | - |
