# Supplementary material for: Costs and scale-up costs of community-based Oral HIV Self-Testing for female sex workers and men who have sex with men in Jakarta and Bali, Indonesia
Source: BMC Health Serv Res. 2024 Jan 22;24:114. doi: 10.1186/s12913-024-10577-0 (PMC10802071; doi:10.1186/s12913-024-10577-0)
Supplement: Supplementary file 2 — Additional file 2: Appendix 2. Cost from Client Perspective. [file 12913_2024_10577_MOESM2_ESM.docx]

**Appendix 2.**

**Results**

**Cost from Client Perspective**

A total of 149 participants from MSM and FSW in Jakarta and Bali who did HIV CBS were recruited. Across area in Jakarta and Bali, clients from KP of FSW and MSM who did HIV CBS were approximately 33 years of age, female (59.1%), single (never married or separated/divorced) (79.2%), had finished secondary level education (57.7%) and employed (94%). Monthly household expenditure was estimated at US$372.17 with most of the clients categorized into 20% richest expenditure based on Indonesia Socio Economic Survey (SUSENAS) in 2021.^1^ Most of client access information and did HIV CBS on the same day (78.5%) and 28.2% did confirmatory test.

In this study, we estimate the client’s average cost of HIV CBS including if the clients screening results reactive and undergo confirmatory test. As per the technical guideline of HIV CBS from Indonesia MOH, we measured the average cost of HIV CBS from the phase of accessing HIV CBS information acquired from peer leaders, self-screening process to confirmation test. We found that across Jakarta and Bali, 78.5% respondents access information and screening at the same day but small proportion of respondent (21.5%) access in two different days. For clients who did 1-day HIV CBS from accessing program information until screening process, the total cost per clients (direct non-medical and indirect cost) ranged from $1.9 to $12.2 (lowest among MSM in Bali and highest among FSW in Jakarta). Total cost per clients for patient who did 2 days HIV CBS were higher compare to 1 days. From these total costs, out of pocket spending to purchase direct non-medical cost (OOP) of clients who did 1-day HIV CBS ranged approximately from $0.1 (3.74% of total costs) (among FSW in Bali) to $9.5 (78.1%) (among FSW in Jakarta). While for clients who did 2-days HIV CBS, OOP were relatively higher, ranged from $0.1 (2.61%) (among FSW in Bali) to $9.9 (70.33%) (among FSW in Jakarta). Whether 1 days or 2 days HIV CBS, both of OOP spending were lowest among MSM in Bali and highest among FSW in Jakarta. We found that there was an average of 28.2% of total clients (ranged from 18.7% to 37.8%) who underwent confirmation test. For clients in Jakarta who did confirmation test, 50% of the expenses were due to non-health care costs followed by health care costs and lost of productivity costs. Details of client characteristics and client costs who did HIV CBS presented in table A3 and table A4.

**References:**

1. Statistics Indonesia (BPS). *Socio Economic Survey (SUSENAS) 2021*. (2021).

**Tabel A3. Characteristics of clients who did HIV Community-based Screening in Jakarta and Bali**

| **Characteristic** | | **Total** | |  | **Jakarta** | |  | **Bali** | | **Mean** |
| --- | --- | --- | --- | --- | --- | --- | --- | --- | --- | --- |
|  |  | **n** | **%** |  | **n** | **%** |  | **n** | **%** |  |
| **Total** | | **149** |  |  | **74** | **49.7** |  | **75** | **50.3** |  |
| Sex | |  |  |  |  |  |  |  |  |  |
|  | Female | 88 | 59.1 |  | 50 | 67.6 |  | 38 | 50.7 |  |
|  | Male | 61 | 40.9 |  | 24 | 32.4 |  | 37 | 49.3 |  |
| Age (years) | |  |  |  |  |  |  |  |  |  |
|  | 17-25 | 38 | 25.5 |  | 15 | 20.3 |  | 23 | 30.7 | 33 years |
|  | 26-45 | 92 | 61.7 |  | 48 | 64.9 |  | 44 | 58.7 |  |
|  | 46-65 | 19 | 12.8 |  | 11 | 14.9 |  | 8 | 10.7 |  |
| Marital status | |  |  |  |  |  |  |  |  |  |
|  | Single (never married) | 64 | 43.0 |  | 29 | 39.2 |  | 35 | 46.7 |  |
|  | Married | 31 | 20.0 |  | 17 | 23.0 |  | 14 | 18.7 |  |
|  | Separated/ Divorced | 54 | 36.2 |  | 28 | 37.8 |  | 26 | 34.7 |  |
| Educational | |  |  |  |  |  |  |  |  |  |
|  | Low education level | 63 | 42.3 |  | 31 | 41.9 |  | 32 | 42.7 |  |
|  | High education level | 86 | 57.7 |  | 43 | 58.1 |  | 43 | 57.3 |  |
| Expenditure | |  |  |  |  |  |  |  |  |  |
|  | 20% poorest | 1 | 0.7 |  | 0 | 0.0 |  | 1 | 1.3 |  |
|  | 20% pooret | 5 | 3.4 |  | 4 | 5.4 |  | 1 | 1.3 |  |
|  | 20% middle | 8 | 5.4 |  | 5 | 6.8 |  | 3 | 4.0 |  |
|  | 20% richer | 23 | 15.4 |  | 14 | 18.9 |  | 9 | 12.0 |  |
|  | 20% richest | 112 | 75.2 |  | 51 | 68.9 |  | 61 | 81.3 |  |
| Working hours | |  |  |  |  |  |  |  |  | 9 |
| Employment Status | |  |  |  |  |  |  |  |  |  |
|  | Employed | 140 | 94.0 |  | 70 | 94.6 |  | 70 | 93.3 |  |
|  | Unemployed | 9 | 6.0 |  | 4 | 5.4 |  | 5 | 6.7 |  |
| HIV Screening services | |  |  |  |  |  |  |  |  |  |
|  | Access information and screening (on the same day) | 117 | 78.5 |  | 50 | 67.6 |  | 67 | 89.3 |  |
|  | Access information and screnning (on different days) | 32 | 21.5 |  | 24 | 32.4 |  | 8 | 10.7 |  |
|  | Confirmation test | 42 | 28.2 |  | 28 | 37.8 |  | 14 | 18.7 |  |

**Tabel A4. Client Cost of HIV Community-based Screening among FSW and MSM in Jakarta and Bali (US$)**

| **Activities** | **Cost** | | **Jakarta (N=74)** | | | | | | |  | **Bali (N=75)** | | | | | | |
| --- | --- | --- | --- | --- | --- | --- | --- | --- | --- | --- | --- | --- | --- | --- | --- | --- | --- |
|  |  |  | **MSM (n=24)** | | |  | **FSW (n=50)** | | |  | **MSM (n=37)** | | |  | **FSW (n=38)** | | |
|  |  |  | **n** | **Total Cost (US$)** | **Mean (US$)** |  | **n** | **Total Cost (US$)** | **Mean (US$)** |  | **n** | **Total Cost (US$)** | **Mean (US$)** |  | **n** | **Total Cost (US$)** | **Mean (US$)** |
| Acces information and screening (on the same day) | Direct non medical cost | |  |  |  |  |  |  |  |  |  |  |  |  |  |  |  |
|  |  | Transport | 6 | 12.64 | 2.11 |  | 21 | 44.74 | 2.13 |  | 5 | 1.31 | 0.26 |  | 9 | 1.18 | 0.13 |
|  |  | Consumption | 2 | 2.69 | 1.35 |  | 6 | 13.81 | 2.30 |  | - | - | - |  | - | - | - |
|  |  | Seluler | 7 | 10.78 | 1.54 |  | 12 | 16.98 | 1.41 |  | 12 | 8.08 | 0.67 |  | - | - | - |
|  |  | Carer (transport & consumption) | 1 | 1.35 | 1.35 |  | 3 | 9.77 | 3.26 |  | 0 | - | - |  | - | - | - |
|  |  | Other | 2 | 0.27 | 0.13 |  | 8 | 3.50 | 0.44 |  | 0 | - | - |  | - | - | - |
|  | Indirect cost | |  |  |  |  |  |  |  |  |  |  |  |  |  |  |  |
|  |  | client loss of income | 11 | 25.55 | 2.32 |  | 36 | 93.00 | 2.58 |  | 29 | 29.37 | 1.01 |  | 33 | 110.20 | 3.34 |
|  |  | Carer loss of income |  |  |  |  | 1 | 0.10 | 0.10 |  |  |  |  |  | 1 | 0.04 | 0.04 |
| **Total Cost (Unit Cost in US$)** | | |  | **53.28** | **8.80** |  |  | **181.90** | **12.22** |  |  | **38.77** | **1.95** |  |  | **111.42** | **3.51** |
|  |  |  |  |  |  |  |  |  |  |  |  |  |  |  |  |  |  |
| Access information | Direct non medical cost | |  |  |  |  |  |  |  |  |  |  |  |  |  |  |  |
|  |  | Transport | 4 | 0.81 | 0.20 |  | 8 | 11.12 | 1.39 |  | - | - | - |  | 1 | 0.03 | 0.03 |
|  |  | Consumption | 3 | 4.04 | 1.35 |  | 7 | 17.52 | 2.50 |  | - | - | - |  | - | - | - |
|  |  | Seluler | 8 | 6.00 | 0.75 |  | 2 | 4.72 | 2.36 |  | 1 | 1.35 | 1.35 |  | - | - | - |
|  |  | Carer | 1 | 2.02 | 2.02 |  | 2 | 3.37 | 1.68 |  | - | - | - |  | - | - | - |
|  |  | Other | 1 | 0.13 | 0.13 |  | - | - | - |  | - | - | - |  | - | - | - |
|  | Indirect cost | |  |  |  |  |  |  |  |  |  |  |  |  |  |  |  |
|  |  | client loss of income | 13 | 24.56 | 1.89 |  | 11 | 50.06 | 4.55 |  | 3 | 0.94 | 0.31 |  | 5 | 5.97 | 1.19 |
| **Total Cost (Unit Cost in US$)** | | |  | **37.57** | **6.34** |  |  | **86.78** | **12.49** |  |  | **2.28** | **1.66** |  |  | **6.00** | **1.23** |
|  |  |  |  |  |  |  |  |  |  |  |  |  |  |  |  |  |  |
| Screening | Direct non medical cost | |  |  |  |  |  |  |  |  |  |  |  |  |  |  |  |
|  |  | Transport | 5 | 4.67 | 0.93 |  | 8 | 10.71 | 1.34 |  | - | - | - |  | 1 | 0.03 | 0.03 |
|  |  | Consumption | 6 | 8.08 | 1.35 |  | 8 | 14.82 | 1.85 |  | - | - | - |  | - | - | - |
|  |  | Seluler | 5 | 6.74 | 1.35 |  | 1 | 3.37 | 3.37 |  | - | - | - |  | - | - | - |
|  |  | Carer | 5 | 8.76 | 1.75 |  | - | - | - |  | - | - | - |  | - | - | - |
|  |  | Other | 1 | 0.07 | 0.07 |  | - | - | - |  | - | - | - |  | - | - | - |
|  | Indirect cost | |  |  |  |  |  |  |  |  |  |  |  |  |  |  |  |
|  |  | client loss of income | 13 | 29.75 | 2.29 |  | 11 | 50.18 | 4.56 |  | 3 | 1.08 | 0.36 |  | 5 | 6.62 | 1.32 |
| **Total Cost (Unit Cost in US$)** | | |  | **58.07** | **7.74** |  |  | **79.08** | **11.12** |  |  | **1.08** | **0.36** |  |  | **6.65** | **1.36** |
|  |  |  |  |  |  |  |  |  |  |  |  |  |  |  |  |  |  |
| Confirmation test | Direct non medical cost | |  |  |  |  |  |  |  |  |  |  |  |  |  |  |  |
|  |  | Transport | 18 | 41.37 | 2.30 |  | 3 | 11.20 | 3.73 |  | 9 | 4.01 | 0.45 |  | 2 | 0.40 | 0.20 |
|  |  | Consumption | 11 | 21.56 | 1.96 |  | 3 | 4.04 | 1.35 |  | - | - | - |  | 1 | 1.35 | 1.35 |
|  |  | Seluler | 4 | 5.39 | 1.35 |  | 5 | 6.06 | 1.21 |  | - | - | - |  | - | - | - |
|  |  | Carer | 6 | 10.78 | 1.80 |  | 3 | 9.77 | 3.26 |  | - | - | - |  | - | - | - |
|  |  | Other | - | - | - |  | 7 | 1.15 | 0.16 |  | 7 | - | - |  | 2 | 0.13 | 0.07 |
|  | Indirect cost | |  |  |  |  |  |  |  |  |  |  |  |  |  |  |  |
|  |  | client loss of income | 18 | 72.91 | 4.05 |  | 9 | 35.25 | 3.92 |  | 9 | 11.48 | 1.28 |  | 4 | 17.80 | 4.45 |
|  | Direct medical cost | |  |  |  |  |  |  |  |  |  |  |  |  |  |  |  |
|  |  | Administration | 16 | 16.17 | 1.01 |  | 10 | 10.11 | 1.01 |  | 7 | 7.75 | 1.11 |  | 2 | 3.37 | 1.68 |
|  |  | Laboratory | - | - | - |  | 1 | 3.37 | 3.37 |  | - | - | - |  | 1 | 7.07 | 7.07 |
|  |  | Medical services | 1 | 1.01 | 1.01 |  | - | - | - |  | - | - | - |  | - | - | - |
|  |  | Medical treatment | 1 | 1.01 | 1.01 |  | - | - | - |  | - | - | - |  | - | - | - |
| **Total Cost (Unit Cost in US$)** | | |  | **170.21** | **14.48** |  |  | **80.95** | **18.01** |  |  | **23.24** | **2.83** |  |  | **30.13** | **14.82** |

We found that across Jakarta and Bali, 78.5% respondents access information and screening at the same day but small proportion of respondent (21.5%) access in two different days. For clients who did 1-day HIV CBS from accessing program information until screening process, the total cost per clients (direct non-medical and indirect cost) ranged from $1.9 to $12.2 (lowest among MSM in Bali and highest among FSW in Jakarta). Total cost per clients for patient who did 2 days HIV CBS were higher compare to 1 days. From these total costs, out of pocket spending to purchase direct non-medical cost (OOP) of clients who did 1-day HIV CBS ranged approximately from $0.1 (3.74% of total costs) (among FSW in Bali) to $9.5 (78.1%) (among FSW in Jakarta). While for clients who did 2-days HIV CBS, OOP were relatively higher, ranged from $0.1 (2.61%) (among FSW in Bali) to $9.9 (70.33%) (among FSW in Jakarta). Whether 1 days or 2 days HIV CBS, both of OOP spending were lowest among MSM in Bali and highest among FSW in Jakarta. We found that there was an average of 28.2% of total clients (ranged from 18.7% to 37.8%) who underwent confirmation test. For clients in Jakarta who did confirmation test, 50% of the expenses were due to non-health care costs followed by health care costs and lost of productivity cost
