# Supplementary material for: Costs and scale-up costs of community-based Oral HIV Self-Testing for female sex workers and men who have sex with men in Jakarta and Bali, Indonesia
Source: BMC Health Serv Res. 2024 Jan 22;24:114. doi: 10.1186/s12913-024-10577-0 (PMC10802071; doi:10.1186/s12913-024-10577-0)
Supplement: Supplementary file 3 — Additional file 3: Appendix 3. Questionnaire for client cost. [file 12913_2024_10577_MOESM3_ESM.docx]

**Appendix 3. Questionnaire for client cost**

**INTERVIEWER :
INTERVIEW PLACE :**

**INTERVIEW DAY/DATE :**

**INTERVIEW TIME :**

**QUESTIONNAIRE – CLIENT COST**

**RESPONDENT IDENTITY**

Filling instructions: fill in the questions below by writing the answers, or tick (√) in the box provided. If you have difficulty filling in the answer or don't understand the question, ask the interviewer.

| **A.** | **SOCIAL DEMOGRAPHIC CHARACTERISTIC OF RESPONDENTS** | |
| --- | --- | --- |
| 1. | ID | ____________________________________________ |
| 2. | Area of residence (district/city) | ____________________________________________ |
| 3. | Gender | Male 1 Female 2 |
| 4. | Age | ………… Year |
| 5. | Last educational certificate: | |
|  | ☐ 1. Didn't go to school/didn't finish elementary school ☐ 2. Elementary school ☐ 3. Middle school ☐ 4. High school ☐ 5. Diploma  ☐ 6. S1 ☐ 7. S2 ☐ 8. S3 | |
| 6. | Current marital status: | |
|  | ☐ not married 0 ☐ married 1 ☐ divorced 2 ☐ husband/wife died 3 | |
| 7. | Number of children | ………… Person |

| **B** | **JOB AND INCOME** | |
| --- | --- | --- |
| 8. | What is your current job? (more than one answer is possible, go to number 12 if the job does not have an income) | |
|  | -------------------------------------------------- -------------------------------------------------- -------------- | |
| 9. | What is your average income each month?  (if you have more than one job, write down the total amount of your income each month)    **IDR** …………….…………………… | |
| 10. | How many hours on average do you work in a day? | …… O'clock |
| 11. | How many days do you work on average in a week? | …… day |
| 12. | To meet your personal needs in **1 month,** do you also get money from other people? Please state in the column below, along with the amount. | |
|  | \| **No** \| **Earn money from** \| **Amount in the last month** \| \| --- \| --- \| --- \| \| **1** \| Parent \| IDR…………………………….. \| \| **2** \| Brother sister \| IDR…………………………….. \| \| **3** \| Another sibling \| IDR…………………………….. \| \| **4** \| Child \| IDR…………………………….. \| \| **5** \| Friend \| IDR…………………………….. \| \| **6** \| Selling personal items \| IDR…………………………….. \| \| **7** \| Borrow from…. \| IDR…………………………….. \| \| **8** \| Others, please specify………. \| IDR…………………………….. \| | |
| 13. | For what purposes and how much do you spend **each month** ? | |
|  | \| **No** \| **Expenditure** \| **Amount in the last month** \| \| --- \| --- \| --- \| \| **1** \| House rental/house installments \| IDR ………………………………………… \| \| **2** \| Electricity \| IDR ………………………………………… \| \| **3** \| Water \| IDR ………………………………………… \| \| **4** \| House phone \| IDR ………………………………………… \| \| **5** \| Transport/gasoline \| IDR ………………………………………… \| \| **6** \| Cell phone credit \| IDR ………………………………………… \| \| **7** \| To eat at home \| IDR ………………………………………… \| \| **8** \| To eat outside the home \| IDR ………………………………………… \| \| **9** \| Entertainment (snacks, etc.) \| IDR………………………………………….. \| \| **10** \| Cigarette \| IDR ………………………………………… \| \| **11** \| Health/see a doctor \| IDR ………………………………………… \| \| **12** \| Drugs \| IDR ………………………………………… \| \| **13** \| Savings \| IDR ………………………………………… \| \| **14** \| Others…………………………(please specify) \| IDR ………………………………………… \| | |
|  |  | |
| 14. | Who lives at home with you currently?  **How to fill in the** following table:   - Circle the number of the person you live with, if it is not on the list, write that person in numbers 7-9 - Write down the person's occupation (including if the job is a housewife, student or not working) and the person's income | |
|  | \| 1. Family members or others \| \| 1. Work \| 1. Total income in one month \| 1. Does it accompany you during counseling, screening and tests? \| 1. Time between PP minutes \| 1. Working time of day (hours) \| 1. Week working time \| \| --- \| --- \| --- \| --- \| --- \| --- \| --- \| --- \| \| 1 \| Father \| ...... \| IDR................................. \| **☐** counseling  **☐** screening  **☐** confirmation test \|  \|  \|  \| \| 2 \| Mother \| ...... \| IDR................................. \| **☐** counseling  **☐** screening  **☐** confirmation test \|  \|  \|  \| \| 3 \| Older brother \| ...... \| IDR................................. \| **☐** counseling  **☐** screening  **☐** confirmation test \|  \|  \|  \| \| 4 \| Younger brother \| ...... \| IDR................................. \| **☐** counseling  **☐** screening  **☐** confirmation test \|  \|  \|  \| \| 5 \| Wife husband \| ...... \| IDR................................. \| **☐** counseling  **☐** screening  **☐** confirmation test \|  \|  \|  \| \| 6 \| Child \| ...... \| IDR................................. \| **☐** counseling  **☐** screening  **☐** confirmation test \|  \|  \|  \| \| 7 \| ...... \| ...... \| IDR................................. \| **☐** counseling  **☐** screening  **☐** confirmation test \|  \|  \|  \| \| 8 \| ...... \| ...... \| IDR................................. \| **☐** counseling  **☐** screening  **☐** confirmation test \|  \|  \|  \| \| 9 \| ...... \| ...... \| IDR................................. \| **☐** counseling  **☐** screening  **☐** confirmation test \|  \|  \|  \| | |

| 15. | \| 1. Non-family members \| \| 1. Work \| 1. Total income in one month \| 1. Does it accompany you during counseling, screening and tests? \| 1. Time between PP minutes \| 1. Working time of day (hours) \| 1. Week working time \| \| --- \| --- \| --- \| --- \| --- \| --- \| --- \| --- \| \| 1 \| Father \| ...... \| IDR..................... \| **☐** counseling  **☐** screening  **☐** confirmation test \|  \|  \|  \| \| 2 \| Mother \| ...... \| IDR..................... \| **☐** counseling  **☐** screening  **☐** confirmation test \|  \|  \|  \| \| 3 \| Older brother \| ...... \| IDR................................. \| **☐** counseling  **☐** screening  **☐** confirmation test \|  \|  \|  \| \| 4 \| Younger brother \| ...... \| IDR................................. \| **☐** counseling  **☐** screening  **☐** confirmation test \|  \|  \|  \| \| 5 \| Wife husband \| ...... \| IDR................................. \| **☐** counseling  **☐** screening  **☐** confirmation test \|  \|  \|  \| \| 6 \| Child \| ...... \| IDR................................. \| **☐** counseling  **☐** screening  **☐** confirmation test \|  \|  \|  \| \| 7 \| ...... \| ...... \| IDR................................. \| **☐** counseling  **☐** screening  **☐** confirmation test \|  \|  \|  \| \| 8 \| ...... \| ...... \| IDR................................. \| **☐** counseling  **☐** screening  **☐** confirmation test \|  \|  \|  \| \| 9 \| ...... \| ...... \| IDR................................. \| **☐** counseling  **☐** screening  **☐** confirmation test \|  \|  \|  \| |
| --- | --- | --- | --- | --- | --- | --- | --- | --- | --- | --- | --- | --- | --- | --- | --- | --- | --- | --- | --- | --- | --- | --- | --- | --- | --- | --- | --- | --- | --- | --- | --- | --- | --- | --- | --- | --- | --- | --- | --- | --- | --- | --- | --- | --- | --- | --- | --- | --- | --- | --- | --- | --- | --- | --- | --- | --- | --- | --- | --- | --- | --- | --- | --- | --- | --- | --- | --- | --- | --- | --- | --- | --- | --- | --- | --- | --- | --- | --- | --- | --- | --- |

| **C** | **PRE-SCREENING COUNSELING COSTS** | |
| --- | --- | --- |
| 16. | Did you get information regarding HIV testing and/or HIV screening face-to-face or online?  …………………………………………………………………………………………. | |
| 17. | If face to face, where do you get information regarding HIV testing and/or HIV screening? (At NGO services, at a health center, at work, or other locations, please specify)  …………………………………………………………………………………………. | |
| 18. | If online, what media do you use to get information regarding HIV testing and/or HIV screening? (website, WhatsApp, Instagram, Facebook, or other media please specify)  …………………………………………………………………………………………. | |
| 19. | If face to face, how do you get to that place? | |
|  | ☐ 1. Walk  ☐ 2. Bicycle  ☐ 3. Private motorbike  ☐ 4. Ojeg  ☐ 5. Private car | ☐ 6. City transportation (angkot)  ☐ 7. Taxis  ☐ 8. Public bus  ☐ 9. *Online Transportation*  ☐ 10. Others, namely... |
| 20. | How long does it take to get there? | …… minute |
| 21. | What is the distance from your house to the facility? ……km | |
| 22 . | How long does it take to get information regarding HIV testing/HIV screening both face to face and online? (including waiting time) ……..minutes/hour | |
| 23 . | To get information regarding HIV testing and/or HIV screening? What is the average cost incurred for (leave blank if not relevant): | |
|  | \| 1. Own round trip transportation \| IDR................ \| \| --- \| --- \| \| 1. Round trip transportation for the person accompanying you \| IDR................ \| \| 1. Registration \| IDR................ \| \| 1. Services for counselors (out of pocket) \| IDR................ \| \| 1. Introductory Meals \| IDR................ \| \| 1. Eat alone \| IDR................ \| \| 1. Lodging \| IDR................ \| \| 1. Credit/internet \| IDR................ \| \|  \|  \| | |

| **D** | **COMMUNITY-BASED HIV SCREENING COSTS** | |
| --- | --- | --- |
| 24. | Do you carry out HIV screening independently with assistance or without assistance?  …………………………………………………………………………………………. | |
| 25. | When do you self-screen for HIV? (a. same day as pre-screening counseling/b. different day)  …………………………………………………………………………………………. | |
| 26. | If on different days, where do you screen for HIV? (in the same place or another place? Please specify if the other place is serviced (NGO, health center health facility, workplace, hangout place, home, etc.)  …………………………………………………………………………………………. | |
| 27 . | If you are in another place and if you are not at home, how do you get to that place? | |
|  | ☐ 1. Walk  ☐ 2. Bicycle  ☐ 3. Private motorbike  ☐ 4. Ojeg  ☐ 5. Private car | ☐ 6. City transportation (angkot)  ☐ 7. Taxis  ☐ 8. Public bus  ☐ 9. *Online Transportation*  ☐ 10. Others, namely... |
| 28 . | How long does it take to get there? | …… minute |
| 29. | What is the distance from your house to the facility? ……km | |
| 30 . | How long does it take to carry out independent HIV screening (including waiting time and results) ……..minutes/hour | |
| 31 . | Wherever you carry out independent HIV screening, what is the average cost for (leave blank if not relevant): | |
|  | \| 1. Own round trip transportation \| IDR................ \| \| --- \| --- \| \| 1. Round trip transportation for the person accompanying you \| IDR................ \| \| 1. Registration \| IDR................ \| \| 1. Services (for counselors/companions) (out of pocket) \| IDR................ \| \| 1. Cost of screening test kit \| IDR…………. \| \| 1. Introductory Meals \| IDR................ \| \| 1. Eat alone \| IDR................ \| \| 1. Lodging \| IDR................ \| \| 1. Credit/internet \| IDR................ \| \| 1. Cost of sending results to lifters/healthcare fans \| IDR………….. \| | |

| **E** | **HIV CONFIRMATION TEST COSTS/HIV TEST COSTS** | |
| --- | --- | --- |
| 32. | When do you self-screen for HIV? (a. same day as screening/b. different day)  …………………………………………………………………………………………. | |
| 33. | If on different days, where do you screen for HIV? (in the same place or another place? Please specify if the other place is serviced (NGO, health center health facility, workplace, hangout place, home, etc.)  …………………………………………………………………………………………. | |
| 3 4. | If you are in another place and if you are not at home, how do you get to that place? | |
|  | ☐ 1. Walk  ☐ 2. Bicycle  ☐ 3. Private motorbike  ☐ 4. Ojeg  ☐ 5. Private car | ☐ 6. City transportation (angkot)  ☐ 7. Taxis  ☐ 8. Public bus  ☐ 9. *Online Transportation*  ☐ 10. Others, namely... |
| 3 5. | How long does it take to get there? | …… minute |
| 3 6. | What is the distance from your house to the facility? ……km | |
| 3 7. | How long does it take to carry out independent HIV screening (including waiting time and results) ……..minutes/hour | |
| 3 8. | Wherever you carry out independent HIV screening, what is the average cost for (leave blank if not relevant): | |
|  | \| 1. Own round trip transportation \| IDR................ \| \| --- \| --- \| \| 1. Round trip transportation for the person accompanying you \| IDR................ \| \| 1. Registration \| IDR................ \| \| 1. Medical treatment \| IDR................ \| \| 1. Laboratory \| IDR................ \| \| 1. Medical services (out of pocket) \| IDR................ \| \| 1. Introductory Meals \| IDR................ \| \| 1. Eat alone \| IDR................ \| \| 1. Lodging \| IDR................ \| \| 1. Credit/internet \| IDR................ \| \|  \|  \| | |
